# IL-10–STAT3-Dependent Transcriptional Regulation in Microglia: Alzheimer’s Disease and Neuroinflammation

**DOI:** 10.3390/biomedicines14040826

**Published:** 2026-04-05

**Authors:** Mi Eun Kim, Jun Sik Lee

**Affiliations:** Immunology Research Lab & BK21-Four Educational Research Group for Age-Associated Disorder Control Technology, Department of biological Science, Chosun University, Gwangju 61452, Republic of Korea; kimme0303@chosun.ac.kr

**Keywords:** IL-10, STAT3, microglia, neuroinflammation

## Abstract

Interleukin-10 (IL-10) is a key immunoregulatory cytokine that suppresses inflammatory gene transcription in myeloid cells through signal transducer and activator of transcription 3 (STAT3). In Alzheimer’s disease and neuroinflammation, microglia express *IL10ra* and exhibit STAT3 Tyr705 phosphorylation following IL-10 stimulation, indicating IL-10 receptor-dependent STAT3 activation. Recent studies demonstrate that IL-10 induces promoter-selective STAT3-dependent transcriptional regulation in microglia through chromatin-associated mechanisms, whereas gp130-dependent cytokines activate STAT3 to induce transcription of defined target genes, including *Socs3* and *Ccl5*. Following IL-10 receptor activation, STAT3 binds regulatory regions of inflammatory genes, including *Il1b, Tnf, Il6*, and *Nlrp3*, with reduced RNA polymerase II and NF-κB binding. IL-10-dependent transcriptional repression involves formation of a nuclear SHIP1–STAT3 complex, localization of histone deacetylase (HDAC)1 and HDAC2 to H3K4me1-enriched enhancer regions, reduced H3K27ac, and decreased chromatin accessibility at regulatory regions of inflammatory genes. IL-10-activated STAT3 induces *Socs3*, which regulates JAK1 and TYK2 activity and STAT3 phosphorylation. Impairment of IL-10 receptor signaling in microglia is associated with increased inflammatory gene expression, enhanced inflammasome-related transcription, demyelination, and amyloid accumulation. This review focuses on IL-10–STAT3-dependent transcriptional regulation in microglia, including receptor signaling, chromatin-associated mechanisms, and disease-associated gene expression in Alzheimer’s disease and neuroinflammation.

## 1. Introduction

Inflammatory gene transcription in microglia during Alzheimer’s disease and neuroinflammation is regulated by cytokine-activated pathways. Signal transducer and activator of transcription 3 (STAT3) is phosphorylated downstream of both pro-inflammatory and immunoregulatory cytokines. Although interleukin (IL)-10 and gp130-dependent cytokines induce STAT3 Tyr705 phosphorylation, they generate different transcriptional responses [1,2]. The mechanisms that determine cytokine-specific STAT3 activity in microglia during Alzheimer’s disease and neuroinflammation are not fully characterized.

IL-10 is an immunoregulatory cytokine that suppresses transcription of *Il1b*, tumor necrosis factor (*Tnf*), *Il6*, and NLR family pyrin domain-containing 3 (*Nlrp3*) in myeloid cells. In peripheral macrophages, IL-10-dependent STAT3 activation is associated with repression of inflammatory gene promoters, and related transcriptional mechanisms have been examined in microglia during Alzheimer’s disease and neuroinflammation. Microglia exhibit greater chromatin organization, enhancer-associated histone modifications, and cytokine receptor expression compared with peripheral myeloid cells [3,4]. Recent genetic, epigenomic, and single-cell studies indicate that IL-10–STAT3 signaling in microglia involves promoter-selective chromatin binding, enhancer-associated histone modification, and cofactor-dependent transcriptional repression. Disease models of autoimmune demyelination and amyloid pathology demonstrate that IL-10 receptor signaling regulates inflammasome gene transcription, debris clearance pathways, and lesion-associated microglial responses [5,6,7].

This review focuses on IL-10–STAT3-dependent transcriptional regulation in microglia in Alzheimer’s disease and neuroinflammation. It describes receptor-mediated STAT3 activation, chromatin-associated mechanisms, and gene-specific transcriptional regulation in microglia. Cytokine-specific STAT3 activity in microglia regulates inflammatory gene transcription and is implicated in demyelinating disease, Alzheimer’s disease, and neuroinflammation.

Studies were selected based on their focus on IL-10–STAT3 signaling in microglia, including experimental models and human data in Alzheimer’s disease and neuroinflammation.

## 2. IL-10–STAT3 Signaling in Microglia During Neuroinflammation

### 2.1. Receptor-Triggered STAT3 Activation and Transcriptional Regulation

IL-10 signals through a heterotetrameric receptor complex composed of two IL-10R1 (IL10RA) and two IL-10R2 (IL10RB) subunits. IL-10R1 mediates ligand recognition, whereas IL-10R2 functions as the signal-transducing subunit required for intracellular signal transduction [8,9]. Ligand binding induces activation of receptor-associated kinases Janus kinase 1 (JAK1), which associates with IL-10R1, and tyrosine kinase 2 (TYK2), which associates with IL-10R2, followed by phosphorylation of tyrosine residues within the IL-10R1 cytoplasmic domain [10,11]. STAT3 phosphorylation at Tyr705 induces homodimerization and nuclear accumulation, leading to binding at γ-activated sequence (GAS) elements within target gene regulatory regions [12,13].

In microglia, STAT3 Tyr705 phosphorylation is induced after IL-10 stimulation [5]. Following IL-10 treatment, STAT3 is enriched at *Il1b*, *Tnf, Il6*, and *Nlrp3* promoters in lipopolysaccharide (LPS)-stimulated myeloid cells. STAT3 binding at these loci is accompanied by reduced RNA polymerase II binding and decreased nuclear factor kappa-light-chain-enhancer of activated B cells (NF-κB) p65 binding [6,14]. IL-10-induced STAT3 activation represses inflammatory gene promoters, whereas IL-6-dependent gp130 signaling induces expression of STAT3 target genes such as Suppressor of cytokine signaling 3 (*Socs3*) and *Ccl5* [15,16]. This promoter-selective transcriptional regulation is associated with formation of a nuclear Src homology 2 (SH2) domain-containing inositol 5′-phosphatase 1 (SHIP1)–STAT3 complex following IL-10 receptor activation. SHIP1 associates with STAT3 and is detected at promoters of inflammatory genes in IL-10-treated cells. Genetic analyses demonstrate that SHIP1 is required for IL-10-mediated reduction in inflammatory gene transcription. Genetic deletion of SHIP1 impairs IL-10-dependent reduction in inflammatory gene transcription without altering IL-6-induced STAT3 signaling [17,18]. These findings identify the SHIP1–STAT3 complex as a determinant of promoter-selective STAT3 activity in IL-10-dependent signaling compared with gp130-mediated STAT3 activation.

### 2.2. STAT3 Chromatin Binding and Epigenetic Regulation in Microglia

**Figure 1:**
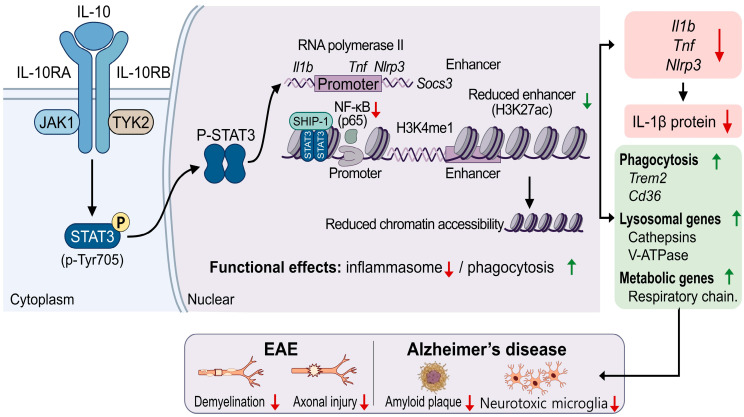
**IL-10–STAT3-dependent chromatin regulation in microglia during neuroinflammation**. IL-10 signaling and STAT3 activation: IL-10 binding to the IL-10 receptor complex (IL-10R1/IL-10R2) activates JAK1 and TYK2, leading to STAT3 phosphorylation at Tyr705. Phosphorylated STAT3 (p-STAT3) dimerizes and translocates to the nucleus. Regulation of inflammatory gene transcription: In the nucleus, p-STAT3 associates with SHIP1 and localizes to regulatory regions of inflammatory genes, including *Il1b, Tnf*, and *Nlrp3*. This localization is associated with reduced RNA polymerase II and NF-κB (p65) binding at gene promoters. At enhancer regions with H3K4me1, IL-10–STAT3 signaling is associated with localization of HDAC1 and HDAC2, reduced H3K27 acetylation, and decreased chromatin accessibility at inflammatory regulatory regions. These chromatin-associated changes are accompanied by reduced transcription of inflammatory genes and decreased IL-1β protein production. Induction of phagocytic and metabolic gene expression: IL-10-activated STAT3 also increases transcription of genes involved in phagocytosis, lysosomal function, and cellular metabolism. These include *Trem2* and *Cd36*, cathepsin family proteases and vacuolar H^+^-ATPase subunits, and mitochondrial respiratory chain components, which contribute to phagocytic uptake, intracellular degradation, and ATP production in microglia.

In microglia, IL-10 stimulation induces STAT3 binding at promoter and enhancer-associated regulatory regions of *Il1b, Il6, Tnf*, and *Nlrp3*. Reduced expression of *Il1b, Il6, Tnf*, and *Nlrp3* is observed in activated microglia exhibiting IL-10-induced STAT3 chromatin binding, and STAT3 binding at regulatory deoxyribonucleic acid (DNA) is associated with decreased inflammatory transcription [6]. *Il10ra* is expressed at higher levels in microglia than in neurons and astrocytes in murine neuroinflammatory models. Conditional deletion of *Il10ra* in CX3CR1-expressing microglia results in increased *Il1b* and *Tnf* expression in brain tissue following inflammatory challenge. These genetic observations establish that IL-10 receptor signaling directly regulates inflammatory gene expression in microglia and provides in vivo support for chromatin-level mechanisms in microglia [19,20]. IL-10-dependent STAT3 binding localizes to regulatory regions exhibiting histone H3 lysine 4 monomethylation (H3K4me1), a modification enriched at enhancer regions within inflammatory gene regulatory regions [21]. IL-10 exposure during inflammatory stimulation reduces enrichment of histone H3 lysine 27 acetylation (H3K27ac) at H3K4me1-enriched enhancers within the *Il1b* and *Tnf* regulatory regions. Reduced H3K27ac is detected at regulatory DNA exhibiting STAT3 binding and is observed at enhancer-associated regions in the absence of changes in total cellular H3K27ac levels [22,23]. Reduced H3K27ac is accompanied by decreased chromatin accessibility at inflammatory regulatory elements under IL-10 exposure. IL-10–STAT3 signaling is associated with reduced H3K27ac and decreased chromatin accessibility at inflammatory enhancer regions through histone deacetylase (HDAC)1 and HDAC2 localization. The chromatin-associated mechanisms of IL-10–STAT3 signaling in CNS microglia are shown in Figure 1.

STAT3 binding at H3K4me1-enriched enhancer regions within the *Il1b* and *Tnf* regulatory loci is associated with localization of HDAC isoforms HDAC1 and HDAC2 under IL-10 exposure [24]. Localization of HDAC1/2 is observed at regions exhibiting reduced H3K27ac enrichment. Inhibition of HDAC activity prevents IL-10-dependent reduction in H3K27ac at *Il1b* and *Tnf* regulatory regions and increases inflammatory gene transcription during activation [25,26]. These findings demonstrate that deacetylase activity is required for modification of enhancer-associated acetylation downstream of IL-10-induced STAT3 binding.

SHIP1 contributes to IL-10-dependent STAT3-mediated chromatin regulation. Genetic deletion of SHIP1 impairs IL-10-dependent reduction in inflammatory gene transcription without altering IL-6-induced STAT3 signaling, indicating that SHIP1 is required for IL-10-specific STAT3 activity at inflammatory regulatory DNA [17]. Together, STAT3 binding, localization of HDAC1/2, reduced H3K27ac enrichment, and decreased accessibility at enhancer-associated regulatory DNA are detected in microglia following IL-10 receptor engagement and are associated with reduced inflammatory gene expression [27].

### 2.3. Cell Type Differences in IL-10–STAT3 Signaling

#### 2.3.1. Microglia

*Il10ra* transcripts are abundantly detected in microglia during experimental autoimmune encephalomyelitis (EAE) and following systemic LPS administration [19,28]. Expression of *Il10ra* provides the IL-10R1 subunit required for assembly of IL-10R1 and IL-10R2 heterotetrameric receptor complexes at the microglial plasma membrane. JAK1 associates with the cytoplasmic domain of IL-10R1 and TYK2 associates with IL-10R2, and ligand binding induces activation of these kinases. Engagement of IL-10R1 and IL-10R2 induces phosphorylation of tyrosine residues within the IL-10R1 cytoplasmic domain. Phosphorylated tyrosine motifs bind the SH2 domain of STAT3, leading to STAT3 phosphorylation at Tyr705. Phosphorylated STAT3 forms dimers and translocates to the nucleus, where it binds regulatory regions of inflammatory genes.

Myeloid-specific deletion of *Il10ra* increases transcription of *Il1b, Tnf*, and *Nos2* in brain-resident CD11b+CD45int microglia during inflammation [29,30]. Deletion of *Il10ra* prevents IL-10-dependent STAT3 phosphorylation and impairs STAT3-mediated repression of *Il1b, Tnf*, and *Nos2* transcription. Deletion of *Il10ra* is accompanied by increased Nos2 transcription in activated microglia, indicating reduced IL-10–STAT3-mediated repression of nitric oxide-associated gene expression.

IL-10 induces STAT3 Tyr705 phosphorylation in microglia and reduces inflammatory mRNA levels, whereas STAT1 phosphorylation is not detected [26]. STAT3 binds regulatory regions of *Il1b, Tnf*, and *Il6*. RNA polymerase II levels at these promoters decrease, and transcript levels are reduced in activated microglia. Deletion of *Stat3* in CX3CR1-expressing microglia increases transcription of *Il1b* and *Tnf* in brain tissue during inflammation. Il10 transcription is present in these microglia [31,32]. Deletion of *Stat3* in microglia increases transcription of *Il1b* and *Tnf*. *Il10* transcription is present in microglia lacking STAT3. IL-10 signaling requires STAT3 to reduce inflammatory gene transcription. Microglial STAT3 binds regulatory regions of inflammatory genes downstream of IL-10 receptor activation. Deletion of *Stat3* results in NF-κB and other pro-inflammatory transcription factors binding these promoters, which increases cytokine transcription.

#### 2.3.2. Neurons

Neurons express low levels of *Il10ra* transcripts in brain tissue and after inflammatory stimulation. Low *Il10ra* expression in neurons results in decreased formation of IL-10 receptor complexes at the plasma membrane. JAK1 and TYK2 activation after ligand binding decreases. STAT3 phosphorylation at Tyr705 is detected at low levels in neurons after IL-10 administration [33]. IL-10 administration induces STAT3 Tyr705 phosphorylation in NeuN-positive neuronal populations at low levels. STAT3 forms dimers and translocates to the nucleus at low levels in neurons. In Iba1-positive microglia, STAT3 accumulates in the nucleus. Neuronal nuclei contain low levels of phospho-STAT3 [34]. Low nuclear STAT3 levels in neurons reduce STAT3 binding to GAS elements within regulatory regions of inflammatory genes. IL-10 exposure reduces transcription of *Il1b*, *Tnf*, and *Il6* in neurons at low levels. Deletion of *Stat3* in neurons does not increase inflammatory cytokine transcription in brain tissue during inflammation. Deletion of Stat3 in myeloid cells increases transcription of these cytokine genes [32,35].

#### 2.3.3. Astrocytes

Astrocytes express *Il10ra* transcripts in brain tissue and after inflammatory stimulation. IL-10 exposure induces STAT3 Tyr705 phosphorylation in astrocytes, and phosphorylated STAT3 translocates to the nucleus. Nuclear STAT3 binds regulatory regions of target genes and modulates transcription [36,37,38].

IL-10 treatment increases transcription of STAT3 target genes, including *Socs3*, in astrocytes. Phosphorylated STAT3 is detected in the nucleus of astrocytes and binds regulatory regions of target genes. *Socs3* transcription in astrocytes is detected at low levels. IL-10 reduces *Il1b* and *Tnf* transcript levels in activated astrocytes [39].

Compared with microglia, astrocytes and neurons show reduced IL10RA expression and decreased STAT3 activation, with reduced transcriptional responses to IL-10.

## 3. IL-10–STAT3-Dependent Transcriptional Regulation in Microglia

### 3.1. Suppression of Inflammatory Gene Transcription

IL-10-activated STAT3 reduces transcription of *Il1b*, *Tnf*, *Il6*, and *Nlrp3* in microglia, with reduced RNA polymerase II at their promoter regions and STAT3 binding at inflammatory gene promoters and enhancers. NF-κB p65 is reduced at inflammatory promoters under IL-10 signaling. IL-10–STAT3 signaling decreases transcription of *Nlrp3* and pro-*Il1b* in activated microglia. Reduced *Nlrp3* mRNA is associated with reduced NLRP3 protein levels. Reduced pro-*Il1b* transcription decreases precursor IL-1β protein levels required for caspase-1-mediated cleavage. In activated microglia, IL-10 exposure is associated with reduced caspase-1 processing and decreased release of mature IL-1β [40,41]. Deletion of microglial *Stat3* similarly increases inflammatory transcripts. Deletion of *Il10ra* or *Stat3* increases *Il1b* and *Tnf* transcription in microglia, indicating decreased IL-10-dependent repression at inflammatory gene promoters [19].

At the chromatin level, IL-10 stimulation is associated with decreased H3K27ac at enhancer regions of *Il1b* and *Tnf*. Reduced H3K27ac is present at genomic regions with STAT3 binding. Inflammatory enhancers show decreased acetylation and reduced chromatin accessibility, whereas total cellular histone acetylation remains unchanged [3,22]. HDAC isoforms HDAC1 and HDAC2 localize to inflammatory regulatory regions during IL-10–STAT3 signaling. Inhibition of HDAC activity prevents loss of H3K27ac at these loci and increases inflammatory gene transcription [42,43].

IL-10-activated STAT3 produces a transcriptional response separate from gp130-dependent STAT3 activation. IL-10 induces nuclear association of STAT3 with SHIP1. SHIP1 is detected at promoters of inflammatory genes after IL-10 receptor engagement. Genetic deletion of SHIP1 impairs IL-10-mediated reduction in *Il1b* and *Tnf* transcription without altering IL-6-induced STAT3 target gene expression. STAT3–SHIP1 complexes are detected at inflammatory gene regulatory regions following IL-10 receptor activation and are associated with decreased *Il1b* and *Tnf* transcription [17,44].

### 3.2. Induction of Immunoregulatory and Phagocytic Genes

IL-10-activated STAT3 induces immunoregulatory and phagocytosis-related genes in microglia through direct promoter binding and transcriptional activation. IL-10 receptor engagement induces STAT3 phosphorylation at Tyr705, leading to nuclear accumulation and binding to γ-activated sequence (GAS) motifs within target gene promoters [28,45]. STAT3 directly induces transcription of *Socs3* in microglia. STAT3 enrichment at the *Socs3* promoter is accompanied by increased RNA polymerase II and increased *Socs3* mRNA levels. SOCS3 protein contains a kinase inhibitory region that associates with JAK1 and TYK2, reducing STAT phosphorylation through inhibition of receptor-associated kinase activity. The SOCS box domain of SOCS3 interacts with components of E3 ubiquitin ligase complexes and promotes ubiquitination of receptor-associated JAK kinases. STAT3-dependent induction of *Socs3* reduces cytokine receptor signaling in microglia.

IL-10–STAT3 signaling promotes transcription of genes required for phagocytic clearance of apoptotic cells, lipid-rich debris, and protein aggregates. STAT3-binding motifs are present within regulatory regions of the triggering receptor expressed on myeloid cells 2 (*Trem2*), and IL-10 increases *Trem2* mRNA and protein expression in microglia. Increased TREM2 expression enhances receptor-mediated phagocytosis of apoptotic neurons, lipid-rich myelin debris, and aggregated proteins [6,46]. IL-10 increases transcription and surface expression of scavenger receptors, including *Cd36* and *Msr1*, and complement receptors such as *Itgam* (CD11b), *C3ar1*, and *C5ar1* in microglia. Increased receptor expression enhances ligand binding and phagocytosis at the plasma membrane [47,48]. IL-10-activated STAT3 increases transcription of genes encoding lysosomal proteases and membrane trafficking proteins involved in intracellular degradation. IL-10 exposure increases expression of cathepsin family members, vesicular trafficking regulators, and subunits of the vacuolar H+-ATPase complex that mediates endosomal acidification. Increased expression of these genes results in enhanced lysosomal acidification and proteolytic activity in microglia [45,49,50].

STAT3-dependent transcription includes genes supporting mitochondrial metabolism. IL-10 increases expression of enzymes involved in the tricarboxylic acid cycle and electron transport chain, including subunits of NADH dehydrogenase (complex I) and cytochrome c oxidase (complex IV). Increased expression of these genes results in increased mitochondrial respiration and ATP production in microglia. Enhanced oxidative phosphorylation increases ATP production for actin remodeling during phagocytosis, vesicular trafficking, and lysosomal fusion. In addition to transcriptional repression, IL-10–STAT3 signaling induces expression of genes involved in phagocytic uptake of apoptotic cells and myelin debris, lysosomal degradation pathways, and mitochondrial oxidative phosphorylation in microglia [51,52,53]. Increased expression of phagocytic receptors and lysosomal enzymes is associated with increased phagocytic uptake and intracellular degradation capacity in microglia, supporting potential therapeutic modulation of IL-10–STAT3 signaling for the regulation of microglial clearance functions [20,54]. IL-10–STAT3 signaling increases transcription of lysosomal genes, including cathepsin family proteases and subunits of the vacuolar H^+^-ATPase complex, which are required for endosomal acidification and degradation of phagocytosed myelin debris and protein aggregates. IL-10-activated STAT3 increases transcription of genes encoding mitochondrial respiratory chain components, supporting oxidative phosphorylation and ATP production in microglia [52,53].

## 4. Molecular Mechanisms of IL-10–STAT3 Signaling in Microglia

### 4.1. SOCS3-Mediated Regulation of STAT3 Signaling

SOCS3 is a transcriptional target of IL-10-activated STAT3 in microglia. STAT3 phosphorylated at Tyr705 translocates to the nucleus and binds GAS motifs within the *Socs3* promoter. *Socs3* mRNA and protein expression increase after IL-10 stimulation. SOCS3 protein contains an N-terminal kinase inhibitory region (KIR), a central SH2 domain, and a C-terminal SOCS box domain. The SH2 domain binds phosphotyrosine residues within cytokine receptor cytoplasmic domains, including phosphorylated motifs on IL-10R1. The kinase inhibitory region associates with the catalytic domains of JAK1 and TYK2. Association of SOCS3 with JAK1 and TYK2 decreases kinase activity and lowers STAT3 phosphorylation [15,55].

Conditional deletion of *Socs3* in myeloid cells, including microglia, increases STAT3 Tyr705 phosphorylation after IL-10 stimulation. Nuclear STAT3 levels increase, and STAT3 binding to inflammatory gene promoters increases. Transcription of *Il1b*, *Tnf*, and Il6 decreases in *Socs3*-deficient cells. SOCS3 regulates STAT3 phosphorylation and nuclear STAT3 binding during IL-10 signaling [56,57,58]. Increased SOCS3 expression decreases nuclear STAT3 levels. STAT3 binding to inflammatory gene promoters decreases. Transcription of *Il1b*, *Tnf*, and *Il6* increases when SOCS3 expression is increased, with reduced STAT3 phosphorylation during IL-10 signaling. Thus, STAT3-induced SOCS3 inhibits JAK1 and TYK2 activity, leading to reduced STAT3 phosphorylation and modulation of anti-inflammatory gene expression in microglia. The SOCS box domain of SOCS3 interacts with Elongin B/C and Cullin5 to form an E3 ubiquitin ligase complex. Through this complex, SOCS3 promotes ubiquitination of receptor-associated JAK kinases. Ubiquitination facilitates degradation or functional inactivation of JAK1 and TYK2, thereby limiting further STAT3 phosphorylation. This mechanism provides a post-translational control step downstream of IL-10 receptor engagement.

Changes in SOCS3 expression alter cytokine transcription in microglia during IL-10 stimulation. Deletion of *Socs3* increases STAT3 Tyr705 phosphorylation and decreases transcription of *Il1b* and *Tnf*. Increased SOCS3 expression decreases STAT3 phosphorylation and increases transcription of these cytokine genes. SOCS3 regulates STAT3 phosphorylation and cytokine gene transcription during IL-10 signaling in microglia [59,60].

### 4.2. SHIP1–STAT3 Regulation of Promoter-Specific Transcription

IL-10 signaling leads to nuclear interaction between STAT3 and SHIP1. STAT3 activation by gp130-dependent cytokines such as IL-6 proceeds independently of SHIP1. SHIP1 contains an N-terminal SH2 domain, a central inositol 5′-phosphatase catalytic domain, and C-terminal proline-rich regions that mediate protein–protein interactions. SHIP1 hydrolyzes phosphatidylinositol (3,4,5)-trisphosphate to phosphatidylinositol (3,4)-bisphosphate in the cytoplasm. During IL-10 stimulation, SHIP1 localizes to the nucleus and binds phosphorylated STAT3. SHIP1 forms a nuclear complex with STAT3 during IL-10 receptor activation. SHIP1 binding does not change STAT3 Tyr705 phosphorylation. STAT3 Tyr705 phosphorylation levels are not altered by SHIP1 binding. STAT3 activated by IL-6 lacks SHIP1 association and binds regulatory regions distinct from those targeted by IL-10-activated STAT3. IL-10-activated STAT3 and gp130-activated STAT3 bind different chromatin regions. Deletion of SHIP1 decreases IL-10-dependent suppression of *Il1b* and *Tnf* transcription. SHIP1 deletion does not alter IL-6-induced STAT3 target gene expression. In SHIP1-deficient cells, STAT3 Tyr705 phosphorylation increases, and STAT3 binding decreases at enhancer-associated regions marked within the *Il1b* and *Tnf* loci. SHIP1 deficiency in microglia decreases IL-10-dependent suppression of *Il1b* and *Tnf* transcripts. SHIP1 regulates inflammatory gene transcription during IL-10 receptor signaling. SHIP1 regulates HDAC1 and HDAC2 localization at promoter and enhancer regions of *Il1b* and *Tnf*. IL-10-dependent STAT3–SHIP1 complexes increase HDAC1 and HDAC2 binding at these loci. SHIP1 deletion decreases HDAC1 and HDAC2 binding and increases histone H3 lysine 27 acetylation at these regions. Increased histone H3 lysine 27 acetylation is associated with increased transcription of *Il1b* and *Tnf* [61,62].

### 4.3. STAT3 Competition with NF-κB and STAT1 at Cytokine Gene Promoters

In central nervous system cells, IL-10-activated STAT3 regulates transcription during NF-κB and STAT1 activation triggered by cytokine and Toll-like receptor (TLR) signaling [63]. TLR stimulation induces nuclear translocation of NF-κB p65. NF-κB p65 binds κB motifs within promoter regions of *Il1b, Tnf*, and *Nlrp3*. NF-κB binding at these promoters increases RNA polymerase II binding and initiates transcription of inflammatory genes. IL-10 signaling increases STAT3 phosphorylation and nuclear STAT3 levels. STAT3 binds regulatory elements within promoters of *Il1b, Tnf*, and *Nlrp3* that also contain κB motifs. STAT3 binding at promoter regions of *Il1b, Tnf*, and *Nlrp3* decreases transcription during NF-κB activation. IL-10 stimulation decreases nuclear NF-κB p65 binding at promoters of *Il1b, Tnf*, and *Nlrp3*, resulting in decreased transcription of these genes. However, continuous TLR signaling increases nuclear NF-κB p65 levels and increases transcription of *Il1b, Tnf*, and *Nlrp3*, reducing the IL-10-induced decrease in transcription [64].

STAT3 and NF-κB bind regulatory regions within inflammatory gene promoters that contain both STAT3-responsive elements and κB motifs. When nuclear NF-κB p65 levels increase, NF-κB binding at these promoters increases and transcription of *Il1b*, *Tnf*, and *Nlrp3* increases despite nuclear STAT3. When nuclear STAT3 levels increase, STAT3 binding at these promoters increases and NF-κB binding decreases, leading to decreased transcription of *Il1b, Tnf*, and *Nlrp3*. Nuclear levels of STAT3 and NF-κB and their binding at these promoters regulate transcription of *Il1b, Tnf*, and *Nlrp3* in activated microglia.

Interferon-γ (IFN-γ) increases STAT1 phosphorylation at Tyr701, resulting in STAT1 dimer formation and nuclear translocation. STAT1 and STAT3 bind related GAS elements within promoters of inflammatory genes. When nuclear STAT1 and STAT3 are both present, STAT1 binding at GAS elements increases and STAT3 binding decreases at these regulatory regions. Increased STAT1 phosphorylation increases STAT1 binding at GAS elements and decreases STAT3 binding at these sites [65]. In microglia exposed to IFN-γ, STAT1 is phosphorylated at Tyr701, forms dimers, and accumulates in the nucleus. Nuclear STAT1 binds GAS elements within promoters of *Il1b* and *Tnf* during IL-10 signaling. STAT1 binding at these sites is associated with reduced STAT3 binding and maintenance of *Il1b* and *Tnf* transcription. STAT1 and STAT3 nuclear levels and promoter binding collectively regulate cytokine gene transcription in microglia during combined IFN-γ and IL-10 signaling [66,67]. IL-10–STAT3 signaling is examined with cytokine pathways including TNF, IL-6, and IFN-γ in the regulation of inflammatory gene transcription in microglia. The molecular and transcriptional mechanisms described above are summarized in Table 1.

## 5. IL-10–STAT3 Signaling in Demyelinating and Neurodegenerative Diseases

### 5.1. EAE

EAE is widely used to examine immune-mediated demyelination and neuroinflammation relevant to multiple sclerosis. Recent investigations demonstrate that IL-10 receptor signaling in microglia directly regulates inflammatory gene transcription, demyelination, axonal injury, and leukocyte infiltration during EAE [70,71]. *Il10ra* expression is detected in CX3CR1-positive microglia during EAE. Conditional deletion of *Il10ra* in myeloid-lineage cells results in increased clinical scores, augmented leukocyte infiltration into the spinal cord, and enhanced demyelination. CNS tissue from *Il10ra*-deficient mice exhibits increased transcription of *Il1b*, *Tnf*, and *Nos2* relative to controls. Microglial IL-10 receptor signaling is associated with reduced axonal damage, including decreased axonal swelling and preservation of axonal integrity, and decreased inflammatory gene expression during autoimmune demyelination [72,73].

STAT3 phosphorylation at Tyr705 is detected in microglia isolated from EAE spinal cords. Myeloid-specific deletion of *Stat3* increases neurological deficit scores and *Il1b* and *Tnf* transcription in CNS tissue. Mice lacking microglial *Stat3* exhibit increased demyelinated lesion area and increased axonal injury, indicating that STAT3 mediates IL-10-dependent repression of inflammatory gene transcription during EAE [5,32].

Inflammasome-related transcripts are regulated by IL-10 receptor signaling during EAE. Increased *Nlrp3* and pro-*Il1b* mRNA levels are detected in spinal cords of *Il10ra*-deficient mice. These changes are accompanied by increased caspase-1 cleavage and mature IL-1β protein in CNS tissue. These findings demonstrate that IL-10–STAT3 signaling regulates transcription of inflammasome components during EAE [68]. In addition to repression of inflammatory genes, IL-10–STAT3 signaling regulates genes involved in debris clearance during EAE. Increased *Trem2* and *Cd36* transcripts are detected in microglia from wild-type mice compared with *Il10ra*-deficient mice. Microglia lacking *Il10ra* exhibit reduced *Trem2* expression and impaired clearance of myelin debris within demyelinated lesions. Impaired debris clearance is associated with increased demyelinated lesion area in spinal cord white matter [74]. Therapeutic administration of IL-10 in experimental models reduces *Il1b* and *Nlrp3* transcription, caspase-1 activation, and axonal damage. These effects are accompanied by increased expression of phagocytic receptors, including *Trem2* and *Cd36*, and improved clearance of myelin debris. These observations support IL-10 receptor signaling as a target for modulation of inflammatory gene transcription and microglial responses in demyelinating disease [29,75,76].

Human data support the involvement of IL-10–STAT3 signaling in demyelinating disease. In postmortem brain tissue from individuals with multiple sclerosis, IL10RA mRNA is detected in microglia within active lesions. Phospho-STAT3 is observed in CD68-positive myeloid cells at lesion borders. Lesions with lower IL10RA expression exhibit higher IL1B and TNF transcript levels. These findings indicate that IL-10 receptor expression in microglia is associated with reduced inflammatory gene transcription in human demyelinating lesions [77,78].

### 5.2. Alzheimer’s Disease and Neurodegeneration

Alzheimer’s disease is characterized by amyloid-β accumulation, tau aggregation, synaptic loss, and progressive cognitive impairment. Alzheimer’s disease is a representative neurodegenerative disease in which IL-10–STAT3-dependent transcriptional regulation in microglia has been examined. Microglial gene expression regulates amyloid clearance and inflammatory mediator production. IL-10 receptor signaling regulates microglial transcription in Alzheimer’s disease models and human brain tissue [79,80]. Single-cell RNA sequencing of human Alzheimer’s disease brain tissue identifies IL10RA transcripts in microglial subsets enriched in amyloid plaque-containing regions. IL10RA-positive microglia exhibit lower IL1B and TNF mRNA levels than low-IL10RA populations. Phospho-STAT3 is detected in IBA1-positive plaque-associated microglia in human brain tissue [81]. In amyloid precursor protein transgenic mice, IL-10 administration increases microglial STAT3 Tyr705 phosphorylation and reduces *Il1b* and *Nlrp3* mRNA levels in cortical tissue. Decreased *Nlrp3* expression is associated with reduced caspase-1 activation and decreased mature IL-1β protein levels [29].

*Trem2* expression is implicated in Alzheimer’s disease, and TREM2 variants are associated with increased disease risk. IL-10 exposure increases *Trem2* transcription in primary microglia. In amyloid-bearing mice, IL-10 receptor deficiency reduces *Trem2* mRNA levels and plaque-associated microglial clustering. Decreased *Trem2* expression is associated with increased amyloid plaque burden and dystrophic neurites in cortical regions [82]. Furthermore, IL-10 signaling increases lysosomal protease and vacuolar H^+^-ATPase expression in microglia in amyloid-bearing mice, enhancing degradation of internalized amyloid-β. STAT3 activation concurrently increases transcription of mitochondrial respiratory chain subunits, oxygen consumption, and ATP production [54,69].

In human cerebrospinal fluid, IL-10 concentrations are inversely associated with IL-1β levels in individuals with mild cognitive impairment and Alzheimer’s disease. Higher cerebrospinal fluid (CSF) IL-10 levels are associated with lower CSF IL-1β concentrations. Brain tissue from individuals with advanced Alzheimer’s disease shows reduced IL10RA expression in cortical microglia compared with controls [83]. Reduced IL10RA expression in microglia and increased *IL1B* expression are observed in Alzheimer’s disease brain tissue, indicating reduced IL-10 receptor signaling.

## 6. Conclusions

IL-10–STAT3 signaling functions as a cytokine-specific transcriptional regulator in microglia during Alzheimer’s disease and neuroinflammation, directing repression of inflammatory gene expression through chromatin-associated mechanisms involving SHIP1 and HDAC-dependent modification of enhancer acetylation. These transcriptional and chromatin-associated mechanisms are associated with the IL-10–STAT3-dependent regulation of inflammatory gene repression in microglia. Experimental models of demyelination and amyloid pathology, together with human tissue analyses, demonstrate that IL-10 receptor signaling regulates inflammasome-related gene transcription, lesion-associated microglial responses, and amyloid uptake and degradation.

Future studies should define the molecular determinants that specify STAT3 binding at inflammatory gene regulatory regions and examine how IL-10-dependent chromatin regulation is altered during disease progression. In addition, characterization of cell type-specific IL-10 receptor signaling in the CNS will provide further insight into transcriptional regulation in neuroinflammation and neurodegeneration. Targeting IL-10–STAT3 signaling in microglia may contribute to the regulation of inflammatory gene transcription and microglial activity in Alzheimer’s disease and neuroinflammation.

## Figures and Tables

**Table 1:** IL-10–STAT3-dependent transcriptional regulation in microglia during Alzheimer’s disease and neuroinflammations.

Molecular Mechanism	Molecular Components	Target Genes	Chromatin/ Molecular Modifications	Functional Effects	Disease Association	Refs.
Receptor signaling	IL10RA, IL10RB, JAK1, TYK2, STAT3 (Tyr705)	*Il1b, Tnf, Il6, Nlrp3*	STAT3 binding at GAS elements; reduced RNA polymerase II binding	Reduced inflammatory gene transcription	Neuroinflammation	[6,8,9,10,11,12,13]
SHIP1–STAT3 regulation	SHIP1–STAT3 nuclear complex	*Il1b, Tnf*	Promoter-selective STAT3 binding	IL-10-specific transcriptional repression	Neuroinflammation	[17,18,44]
Enhancer acetylation control	STAT3, HDAC1, HDAC2	*Il1b, Tnf*	Reduced H3K27ac at H3K4me1-enriched enhancers; reduced chromatin accessibility	Reduced inflammatory gene transcription	Neuroinflammation	[21,22,23,24,25,26]
SOCS3-mediated inhibition	SOCS3 (KIR, SH2, SOCS box domains)	*Il1b, Tnf, Il6*	Reduced STAT3 phosphorylation through JAK1 and TYK2 regulation	Regulation of cytokine gene transcription	Neuroinflammation	[55,56,57,58,59,60]
Inflammasome regulation	STAT3, IL-10R	*Nlrp3, pro-Il1b*	Reduced RNA Pol II binding; decreased caspase-1 cleavage	Reduced mature IL-1β production	EAE, Alzheimer’s disease	[40,41,68]
Phagocytic receptor induction	STAT3, TREM2, CD36	*Trem2, Cd36*	STAT3 binding at regulatory regions	Increased debris clearance; amyloid uptake	EAE, Alzheimer’s disease	
Lysosomal gene induction	Cathepsins, V-ATPase subunits	*Lysosomal protease genes*	Increased transcription of degradation-associated genes	Enhanced amyloid degradation	Alzheimer’s disease	[45,49,50,54,69]
Metabolic gene induction	Mitochondrial respiratory chain subunits	*ETC genes*	STAT3-dependent transcription	Increased oxygen consumption and ATP production	Alzheimer’s disease	[51,52,53,54,69]

## Data Availability

No new data were created or analyzed in this study.

## References

[1] Burmeister A.R., Marriott I. (2018). The Interleukin-10 Family of Cytokines and Their Role in the CNS. Front. Cell. Neurosci..

[2] Samad M.A., Ahmad I., Hasan A., Alhashmi M.H., Ayub A., Al-Abbasi F.A., Kumer A., Tabrez S. (2025). STAT3 Signaling Pathway in Health and Disease. MedComm.

[3] Mishra B., Bachu M., Yuan R., Wingert C., Chaudhary V., Brauner C., Bell R., Ivashkiv L.B. (2025). IL-10 targets IRF transcription factors to suppress IFN and inflammatory response genes by epigenetic mechanisms. Nat. Immunol..

[4] Troutman T.D., Kofman E., Glass C.K. (2021). Exploiting dynamic enhancer landscapes to decode macrophage and microglia phenotypes in health and disease. Mol. Cell.

[5] Lu H.C., Kim S., Steelman A.J., Tracy K., Zhou B., Michaud D., Hillhouse A.E., Konganti K., Li J. (2020). STAT3 signaling in myeloid cells promotes pathogenic myelin-specific T cell differentiation and autoimmune demyelination. Proc. Natl. Acad. Sci. USA.

[6] Hutchins A.P., Diez D., Miranda-Saavedra D. (2013). The IL-10/STAT3-mediated anti-inflammatory response: Recent developments and future challenges. Brief. Funct. Genom..

[7] Gordon H., Gan D., Dolojan A., Dennen J., Hoover C.A., Koh Z.M., Chau K., Avalos Arceo R., Cavanaugh C., Li J. (2025). Genetic dissection of microglia cannibalism reveals an IL10 signaling axis controls microglia lifespan. bioRxiv.

[8] Shouval D.S., Ouahed J., Biswas A., Goettel J.A., Horwitz B.H., Klein C., Muise A.M., Snapper S.B. (2014). Interleukin 10 receptor signaling: Master regulator of intestinal mucosal homeostasis in mice and humans. Adv. Immunol..

[9] Shouval D.S., Biswas A., Goettel J.A., McCann K., Conaway E., Redhu N.S., Mascanfroni I.D., Al Adham Z., Lavoie S., Ibourk M. (2014). Interleukin-10 receptor signaling in innate immune cells regulates mucosal immune tolerance and anti-inflammatory macrophage function. Immunity.

[10] Walter M.R. (2014). The molecular basis of IL-10 function: From receptor structure to the onset of signaling. Interleukin-10 in Health and Disease.

[11] Wilbers R.H.P., van Raaij D.R., Westerhof L.B., Bakker J., Smant G., Schots A. (2017). Re-evaluation of IL-10 signaling reveals novel insights on the contribution of the intracellular domain of the IL-10R2 chain. PLoS ONE.

[12] Hsia H.C., Hutti J.E., Baldwin A.S. (2017). Cytosolic DNA Promotes Signal Transducer and Activator of Transcription 3 (STAT3) Phosphorylation by TANK-binding Kinase 1 (TBK1) to Restrain STAT3 Activity. J. Biol. Chem..

[13] Zouein F.A., Altara R., Chen Q., Lesnefsky E.J., Kurdi M., Booz G.W. (2015). Pivotal Importance of STAT3 in Protecting the Heart from Acute and Chronic Stress: New Advancement and Unresolved Issues. Front. Cardiovasc. Med..

[14] Hutchins A.P., Takahashi Y., Miranda-Saavedra D. (2015). Genomic analysis of LPS-stimulated myeloid cells identifies a common pro-inflammatory response but divergent IL-10 anti-inflammatory responses. Sci. Rep..

[15] Cevey A.C., Penas F.N., Alba Soto C.D., Mirkin G.A., Goren N.B. (2019). IL-10/STAT3/SOCS3 Axis Is Involved in the Anti-inflammatory Effect of Benznidazole. Front. Immunol..

[16] Silver J.S., Hunter C.A. (2010). gp130 at the nexus of inflammation, autoimmunity, and cancer. J. Leukoc. Biol..

[17] Chamberlain T.C., Cheung S.T., Yoon J.S.J., Ming-Lum A., Gardill B.R., Shakibakho S., Dzananovic E., Ban F., Samiea A., Jawanda K. (2020). Interleukin-10 and Small Molecule SHIP1 Allosteric Regulators Trigger Anti-inflammatory Effects through SHIP1/STAT3 Complexes. iScience.

[18] Safari F., Yeoh W.J., Perret-Gentil S., Klenke F., Dolder S., Hofstetter W., Krebs P. (2024). SHIP1 deficiency causes inflammation-dependent retardation in skeletal growth. Life Sci. Alliance.

[19] Shemer A., Scheyltjens I., Frumer G.R., Kim J.S., Grozovski J., Ayanaw S., Dassa B., Van Hove H., Chappell-Maor L., Boura-Halfon S. (2020). Interleukin-10 Prevents Pathological Microglia Hyperactivation following Peripheral Endotoxin Challenge. Immunity.

[20] Bido S., Nannoni M., Muggeo S., Gambare D., Ruffini G., Bellini E., Passeri L., Iaia S., Luoni M., Provinciali M. (2024). Microglia-specific IL-10 gene delivery inhibits neuroinflammation and neurodegeneration in a mouse model of Parkinson’s disease. Sci. Transl. Med..

[21] Local A., Huang H., Albuquerque C.P., Singh N., Lee A.Y., Wang W., Wang C., Hsia J.E., Shiau A.K., Ge K. (2018). Identification of H3K4me1-associated proteins at mammalian enhancers. Nat. Genet..

[22] Conaway E.A., de Oliveira D.C., McInnis C.M., Snapper S.B., Horwitz B.H. (2017). Inhibition of Inflammatory Gene Transcription by IL-10 Is Associated with Rapid Suppression of Lipopolysaccharide-Induced Enhancer Activation. J. Immunol..

[23] Zheng Z., Huang G., Gao T., Huang T., Zou M., Zou Y., Duan S. (2020). Epigenetic Changes Associated with Interleukin-10. Front. Immunol..

[24] Chen S., Saeed A., Liu Q., Jiang Q., Xu H., Xiao G.G., Rao L., Duo Y. (2023). Macrophages in immunoregulation and therapeutics. Signal Transduct. Target. Ther..

[25] Meleady L., Towriss M., Kim J., Bacarac V., Dang V., Rowland M.E., Ciernia A.V. (2023). Histone deacetylase 3 regulates microglial function through histone deacetylation. Epigenetics.

[26] Lai T.H., Ozer H.G., Gasparini P., Nigita G., Distefano R., Yu L., Ravikrishnan J., Yilmaz S., Gallegos J., Shukla S. (2023). HDAC1 regulates the chromatin landscape to control transcriptional dependencies in chronic lymphocytic leukemia. Blood Adv..

[27] Surace A.E.A., Hedrich C.M. (2019). The Role of Epigenetics in Autoimmune/Inflammatory Disease. Front. Immunol..

[28] Gravel M., Beland L.C., Soucy G., Abdelhamid E., Rahimian R., Gravel C., Kriz J. (2016). IL-10 Controls Early Microglial Phenotypes and Disease Onset in ALS Caused by Misfolded Superoxide Dismutase 1. J. Neurosci..

[29] Porro C., Cianciulli A., Panaro M.A. (2020). The Regulatory Role of IL-10 in Neurodegenerative Diseases. Biomolecules.

[30] Laffer B., Bauer D., Wasmuth S., Busch M., Jalilvand T.V., Thanos S., Meyer Zu Horste G., Loser K., Langmann T., Heiligenhaus A. (2019). Loss of IL-10 Promotes Differentiation of Microglia to a M1 Phenotype. Front. Cell. Neurosci..

[31] Yun J.H., Lee D.H., Jeong H.S., Kim H.S., Ye S.K., Cho C.H. (2021). STAT3 activation in microglia exacerbates hippocampal neuronal apoptosis in diabetic brains. J. Cell. Physiol..

[32] Zheng Z.V., Chen J., Lyu H., Lam S.Y.E., Lu G., Chan W.Y., Wong G.K.C. (2022). Novel role of STAT3 in microglia-dependent neuroinflammation after experimental subarachnoid haemorrhage. Stroke Vasc. Neurol..

[33] Verma R., Balakrishnan L., Sharma K., Khan A.A., Advani J., Gowda H., Tripathy S.P., Suar M., Pandey A., Gandotra S. (2016). A network map of Interleukin-10 signaling pathway. J. Cell Commun. Signal..

[34] Guillot-Sestier M.V., Doty K.R., Gate D., Rodriguez J., Leung B.P., Rezai-Zadeh K., Town T. (2015). Il10 deficiency rebalances innate immunity to mitigate Alzheimer-like pathology. Neuron.

[35] Park D.J., Kim K.E., Shin H.J., An H.S., Sun Y., Oh J., Roh G.S. (2025). Myeloid-specific STAT3 deletion modulates molecular activation of hippocampal microglia without morphological remodeling. Biochem. Biophys. Res. Commun..

[36] Carlini V., Noonan D.M., Abdalalem E., Goletti D., Sansone C., Calabrone L., Albini A. (2023). The multifaceted nature of IL-10: Regulation, role in immunological homeostasis and its relevance to cancer, COVID-19 and post-COVID conditions. Front. Immunol..

[37] Cekanaviciute E., Buckwalter M.S. (2016). Astrocytes: Integrative Regulators of Neuroinflammation in Stroke and Other Neurological Diseases. Neurotherapeutics.

[38] O’Neil S.M., Hans E.E., Jiang S., Wangler L.M., Godbout J.P. (2022). Astrocyte immunosenescence and deficits in interleukin 10 signaling in the aged brain disrupt the regulation of microglia following innate immune activation. Glia.

[39] Saraiva M., Vieira P., O’Garra A. (2020). Biology and therapeutic potential of interleukin-10. J. Exp. Med..

[40] Pan Y., Chen X.Y., Zhang Q.Y., Kong L.D. (2014). Microglial NLRP3 inflammasome activation mediates IL-1β-related inflammation in prefrontal cortex of depressive rats. Brain Behav. Immun..

[41] Sun Y., Ma J., Li D., Li P., Zhou X., Li Y., He Z., Qin L., Liang L., Luo X. (2019). Interleukin-10 inhibits interleukin-1β production and inflammasome activation of microglia in epileptic seizures. J. Neuroinflamm..

[42] Stanfield B.A., Purves T., Palmer S., Sullenger B., Welty-Wolf K., Haines K., Agarwal S., Kasotakis G. (2021). IL-10 and class 1 histone deacetylases act synergistically and independently on the secretion of proinflammatory mediators in alveolar macrophages. PLoS ONE.

[43] Kelly R.D.W., Stengel K.R., Chandru A., Johnson L.C., Hiebert S.W., Cowley S.M. (2023). Histone Deacetylases (HDACs) maintain expression of the pluripotent gene network via recruitment of RNA polymerase II to coding and non-coding loci. bioRxiv.

[44] Chan C.S., Ming-Lum A., Golds G.B., Lee S.J., Anderson R.J., Mui A.L. (2012). Interleukin-10 inhibits lipopolysaccharide-induced tumor necrosis factor-α translation through a SHIP1-dependent pathway. J. Biol. Chem..

[45] Hu D., Wan L., Chen M., Caudle Y., LeSage G., Li Q., Yin D. (2014). Essential role of IL-10/STAT3 in chronic stress-induced immune suppression. Brain Behav. Immun..

[46] Zhang N., Ji Q., Chen Y., Wen X., Shan F. (2024). TREM2 deficiency impairs the energy metabolism of Schwann cells and exacerbates peripheral neurological deficits. Cell Death Dis..

[47] Yi S., Jiang X., Tang X., Li Y., Xiao C., Zhang J., Zhou T. (2020). IL-4 and IL-10 promotes phagocytic activity of microglia by up-regulation of TREM2. Cytotechnology.

[48] Li Q., Lan X., Han X., Durham F., Wan J., Weiland A., Koehler R.C., Wang J. (2021). Microglia-derived interleukin-10 accelerates post-intracerebral hemorrhage hematoma clearance by regulating CD36. Brain Behav. Immun..

[49] Liu B., Palmfeldt J., Lin L., Colaco A., Clemmensen K.K.B., Huang J., Xu F., Liu X., Maeda K., Luo Y. (2018). STAT3 associates with vacuolar H^+^-ATPase and regulates cytosolic and lysosomal pH. Cell Res..

[50] Chen Y.Y., Liu C.X., Liu H.X., Wen S.Y. (2025). The Emerging Roles of Vacuolar-Type ATPase-Dependent Lysosomal Acidification in Cardiovascular Disease. Biomolecules.

[51] Lahiri T., Brambilla L., Andrade J., Askenazi M., Ueberheide B., Levy D.E. (2021). Mitochondrial STAT3 regulates antioxidant gene expression through complex I-derived NAD in triple negative breast cancer. Mol. Oncol..

[52] Carbognin E., Betto R.M., Soriano M.E., Smith A.G., Martello G. (2016). Stat3 promotes mitochondrial transcription and oxidative respiration during maintenance and induction of naive pluripotency. EMBO J..

[53] Fumagalli M., Lombardi M., Gressens P., Verderio C. (2018). How to reprogram microglia toward beneficial functions. Glia.

[54] Liu H., Zhou Y.C., Song W. (2021). Involvement of IL-10R/STAT3 pathway in amyloid β clearance by microlgia in Alzheimer’s disease. Int. Immunopharmacol..

[55] Keewan E., Matlawska-Wasowska K. (2021). The Emerging Role of Suppressors of Cytokine Signaling (SOCS) in the Development and Progression of Leukemia. Cancers.

[56] Carow B., Rottenberg M.E. (2014). SOCS3, a Major Regulator of Infection and Inflammation. Front. Immunol..

[57] Kaminska B., Mota M., Pizzi M. (2016). Signal transduction and epigenetic mechanisms in the control of microglia activation during neuroinflammation. Biochim. Biophys. Acta.

[58] Ji X.C., Shi Y.J., Zhang Y., Chang M.Z., Zhao G. (2020). Reducing Suppressors of Cytokine Signaling-3 (SOCS3) Expression Promotes M2 Macrophage Polarization and Functional Recovery After Intracerebral Hemorrhage. Front. Neurol..

[59] Kershaw N.J., Laktyushin A., Nicola N.A., Babon J.J. (2014). Reconstruction of an active SOCS3-based E3 ubiquitin ligase complex in vitro: Identification of the active components and JAK2 and gp130 as substrates. Growth Factors.

[60] Dong R., Huang R., Wang J., Liu H., Xu Z. (2021). Effects of Microglial Activation and Polarization on Brain Injury After Stroke. Front. Neurol..

[61] Terzioglu G., Young-Pearse T.L. (2023). Microglial function, INPP5D/SHIP1 signaling, and NLRP3 inflammasome activation: Implications for Alzheimer’s disease. Mol. Neurodegener..

[62] Chen J.S., Wang H.K., Su Y.T., Ho Y.C., Liang C.L., Lee Y.K., Chu T.H., Lin Y.S., Wu C.C. (2025). HDAC1 dysregulation promotes pro-inflammatory microglial activation and aggravates post-stroke neuroinflammation. Ann. Med..

[63] Nicolas C.S., Amici M., Bortolotto Z.A., Doherty A., Csaba Z., Fafouri A., Dournaud P., Gressens P., Collingridge G.L., Peineau S. (2013). The role of JAK-STAT signaling within the CNS. JAK-STAT.

[64] Alatshan A., Benko S. (2021). Nuclear Receptors as Multiple Regulators of NLRP3 Inflammasome Function. Front. Immunol..

[65] Tolomeo M., Cavalli A., Cascio A. (2022). STAT1 and Its Crucial Role in the Control of Viral Infections. Int. J. Mol. Sci..

[66] Butturini E., Carcereri de Prati A., Mariotto S. (2020). Redox Regulation of STAT1 and STAT3 Signaling. Int. J. Mol. Sci..

[67] Sarma U., Maiti M., Nair A., Bhadange S., Bansode Y., Srivastava A., Saha B., Mukherjee D. (2021). Regulation of STAT3 signaling in IFNγ and IL10 pathways and in their cross-talk. Cytokine.

[68] Shao B.Z., Xu Z.Q., Han B.Z., Su D.F., Liu C. (2015). NLRP3 inflammasome and its inhibitors: A review. Front. Pharmacol..

[69] Lau S.F., Fu A.K.Y., Ip N.Y. (2021). Cytokine signaling convergence regulates the microglial state transition in Alzheimer’s disease. Cell. Mol. Life Sci..

[70] Dubik M., Marczynska J., Morch M.T., Webster G., Jensen K.N., Wlodarczyk A., Khorooshi R., Owens T. (2021). Innate Signaling in the CNS Prevents Demyelination in a Focal EAE Model. Front. Neurosci..

[71] Orian J.M. (2025). A New Perspective on Mechanisms of Neurodegeneration in Experimental Autoimmune Encephalomyelitis and Multiple Sclerosis: The Early and Critical Role of Platelets in Neuro/Axonal Loss. J. Neuroimmune Pharmacol..

[72] Orecchioni M., Wolf D., Suryawanshi V., Winkels H., Kobiyama K., Makings J., Kiosses W.B., Ley K. (2022). Deleting interleukin-10 from myeloid cells exacerbates atherosclerosis in Apoe^−/−^ mice. Cell Mol. Life Sci..

[73] Seifert H.A., Gerstner G., Kent G., Vandenbark A.A., Offner H. (2019). Estrogen-induced compensatory mechanisms protect IL-10-deficient mice from developing EAE. J. Neuroinflammation.

[74] Wu Z., Yu S., Hu Y., Hu X., Yang L., Jiang Y., Zhang M., Mu Y., Li Z., Yao F. (2026). TREM2 Facilitates Myelin Debris Clearance but Exacerbates Chronic Inflammation and Fibrosis After Spinal Cord Injury. CNS Neurosci. Ther..

[75] Li J., Wang P., Zhou T., Jiang W., Wu H., Zhang S., Deng L., Wang H. (2023). Neuroprotective effects of interleukin 10 in spinal cord injury. Front. Mol. Neurosci..

[76] Gao Y., Tu D., Yang R., Chu C.H., Hong J.S., Gao H.M. (2020). Through Reducing ROS Production, IL-10 Suppresses Caspase-1-Dependent IL-1β Maturation, thereby Preventing Chronic Neuroinflammation and Neurodegeneration. Int. J. Mol. Sci..

[77] Luo C., Jian C., Liao Y., Huang Q., Wu Y., Liu X., Zou D., Wu Y. (2017). The role of microglia in multiple sclerosis. Neuropsychiatr. Dis. Treat..

[78] Bugbee E., Wang A.A., Gommerman J.L. (2023). Under the influence: Environmental factors as modulators of neuroinflammation through the IL-10/IL-10R axis. Front. Immunol..

[79] Wang Z., Zhang L., Qin C. (2025). Alzheimer’s disease pathogenesis: Standing at the crossroad of lipid metabolism and immune response. Mol. Neurodegener..

[80] Miao J., Ma H., Yang Y., Liao Y., Lin C., Zheng J., Yu M., Lan J. (2023). Microglia in Alzheimer’s disease: Pathogenesis, mechanisms, and therapeutic potentials. Front. Aging Neurosci..

[81] Sun N., Victor M.B., Park Y.P., Xiong X., Scannail A.N., Leary N., Prosper S., Viswanathan S., Luna X., Boix C.A. (2023). Human microglial state dynamics in Alzheimer’s disease progression. Cell.

[82] Feiten A.F., Dahm K., Schlepckow K., van Lengerich B., Suh J.H., Reifschneider A., Wefers B., Bartos L.M., Wind-Mark K., de Weerd L. (2026). TREM2 expression level is critical for microglial state, metabolic capacity and efficacy of TREM2 agonism. Nat. Commun..

[83] Desiato G., Bosco P., Cintoli S., Biagi L., Braschi C., Del Nero C., Minichiello I., Noale M., Faggiani E., Rossi A. (2025). An inflammatory fingerprint in mild cognitively impaired patients is reversed by physical and cognitive training. Brain Behav. Immun. Health.

